# Ascites production and prognosis after ventriculoperitoneal shunt for diffuse midline gliomas in children: A case series

**DOI:** 10.1097/MD.0000000000039977

**Published:** 2024-10-04

**Authors:** Chunxia Huang, Xubin Chai, Yunpeng Han, Keyuan Lai, Yuanyang Ye, Shaoqiang Xu

**Affiliations:** a Department of Clinical Laboratory, Guangdong Sanjiu Brain Hospital, Guangzhou, China; b State Key Laboratory of Brain and Cognitive Science, Institute of Biophysics, Chinese Academy of Sciences, Beijing, China; c Department of Graduate School, University of Chinese Academy of Sciences, Beijing, China.

**Keywords:** cerebrospinal fluid cytology, diffuse midline glioma, intra-abdominal metastasis, ventriculoperitoneal shunt

## Abstract

**Rationale:**

DMG is a highly invasive and lethal type of brain tumor. As these tumors progress, they often compromise the CSF circulation, leading to hydrocephalus. Ventriculoperitoneal shunt (VPS) is commonly employed to manage hydrocephalus; however, the complication of VPS-induced ascites, particularly in the presence of tumor cells, is a significant concern that merits attention.

**Patient concerns:**

This case series details 3 pediatric patients diagnosed with brainstem DMG harboring the H3 K27M mutation. Each developed hydrocephalus underwent VPS insertion. Post-operatively, all patients developed carcinomatous ascites with tumor cells detected within the ascitic fluid.

**Diagnoses:**

All 3 patients were diagnosed with intra-abdominal metastasis of DMG H3K27M mutant cancer cells, each presenting with distinct complications.

**Interventions:**

Initially, the patients’ primary head tumors responded to treatment, and their hydrocephalus resolved. However, some time after discharge, each patient developed malignant ascites and received palliative chemotherapy to control symptoms and improve quality of life.

**Outcomes:**

Despite the interventions, all 3 patients died within 1 month of developing malignant ascites, with central respiratory failure being the direct cause of death.

**Lessons:**

These cases underscore the importance of continuous monitoring of both the CSF and ascitic fluid in patients with gliomas. Regular assessments of biochemical composition, cytology, and other diagnostic tests are crucial for early detection of disease progression. This proactive approach facilitates timely clinical judgment and intervention, potentially improving patient outcomes.

## 1. Introduction

Diffuse midline glioma (DMG) in children is an extremely aggressive brain tumor characterized by a nearly 100% mortality rate^[1]^ and a median survival of approximately 9 months.^[2,3]^ Missense mutations in histone H3 isoforms, particularly the H3K27M mutation, are associated with poorer prognosis and reduced response to treatment.^[4]^ Disease progression frequently results in hydrocephalus, for which a ventriculoperitoneal shunt (VPS) remains the preferred intervention.^[5,6]^ Although extranodal dissemination through the VPS is exceptionally rare in DMG, the possibility of tumor spread following the procedure warrants serious consideration.^[7,8]^

Here, we present 3 pediatric cases of brainstem DMG with H3K27M mutation, all of whom developed hydrocephalus and underwent VPS surgery. Post-operatively, each patient developed ascites containing tumor cells. These cases highlight the importance of long-term monitoring and regular cerebrospinal fluid (CSF) analysis. Morphological examination of cells in both the CSF and ascitic fluid may serve as a preventative measure after VPS insertion.

## 2. Case report

### 2.1. Case 1

**Figure 1.**
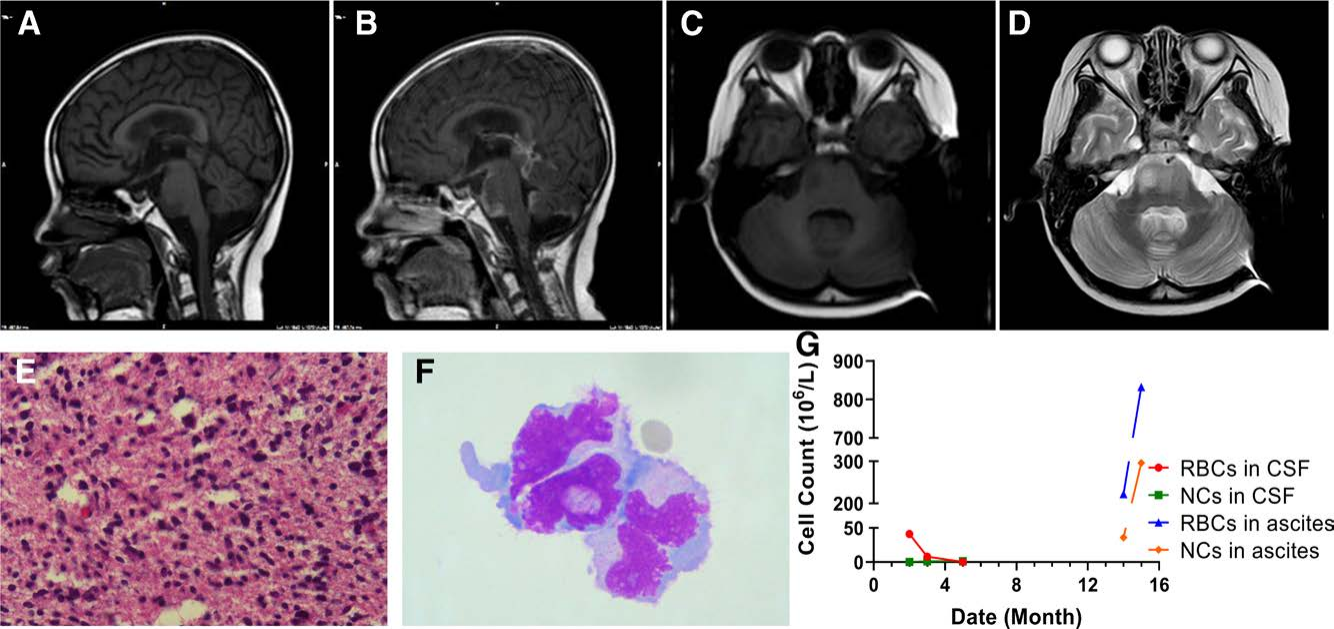
Case 1 (A) Sagittal T1 weighted image. (B) Sagittal T1 contrast-enhanced image. (C) Axial T1 weighted image. (D) Axial T2 weighted image. The lesion semi-circumscribed the adjacent basilar artery and mild postcontrast enhancing. (E) H&E of the cranial stereotactic biopsy. (F) Wright–Giemsa staining of the cells in CSF. (G) Trends of red blood cells (RBCs) and nucleated cells (NCs) in CSF and ascites. CSF = cerebrospinal fluid, NCs = nucleated cells, RBCs = red blood cells.

A 7-year-old male presented to another hospital with persistent nausea and vomiting that was unresponsive to symptomatic treatment. Cranial magnetic resonance imaging (MRI) revealed a poorly defined cystic lesion in the pons, measuring 26 × 31 × 25 mm, characterized by T2 hyperintensity and T1 hypointensity, with heterogeneous internal enhancement (Fig. 1A–D). Histological evaluation confirmed astrocytic glioma (Fig. 1E), and immunohistochemical analysis identified an H3F3A K27M mutation. A diagnosis of the DMG H3K27M-mutant, WHO grade IV) was established. The patient was transferred to our hospital and treated with temozolomide and nitolizumab with no significant clinical improvement. On the 23rd day post-biopsy, cranial MRI indicated secondary hydrocephalus, prompting the discontinuation of chemotherapy and placement of an external ventricular drain. One month later, the drain was converted to a left VPS at Beijing Tiantan Hospital. After hydrocephalus was resolved, chemotherapy was resumed, achieving a 3-month progression-free survival. Subsequently, glioma cells were detected in the CSF (Fig. 1F) and MRI revealed a nodular rim-enhancing mass in the suprasellar cistern. Gamma knife treatment was administered, which resulted in a significant improvement in symptoms. The patient was discharged on analgesics and prophylactic antibiotics.

Thirteen months after the initial diagnosis (and 12 months after VPS insertion), the patient was readmitted with pneumonia and ascites. Temozolomide was administered, and ascitic fluid drainage revealed numerous red blood cells (RBCs) and nucleated cells (NCs; Fig. 1G). Although no tumor mass was identified on abdominal ultrasonography, the possibility of tumor metastasis could not be excluded. Despite further chemotherapy, the patient succumbed to central respiratory failure within 1 month of the development of malignant ascites.

### 2.2. Case 2

**Figure 2.**
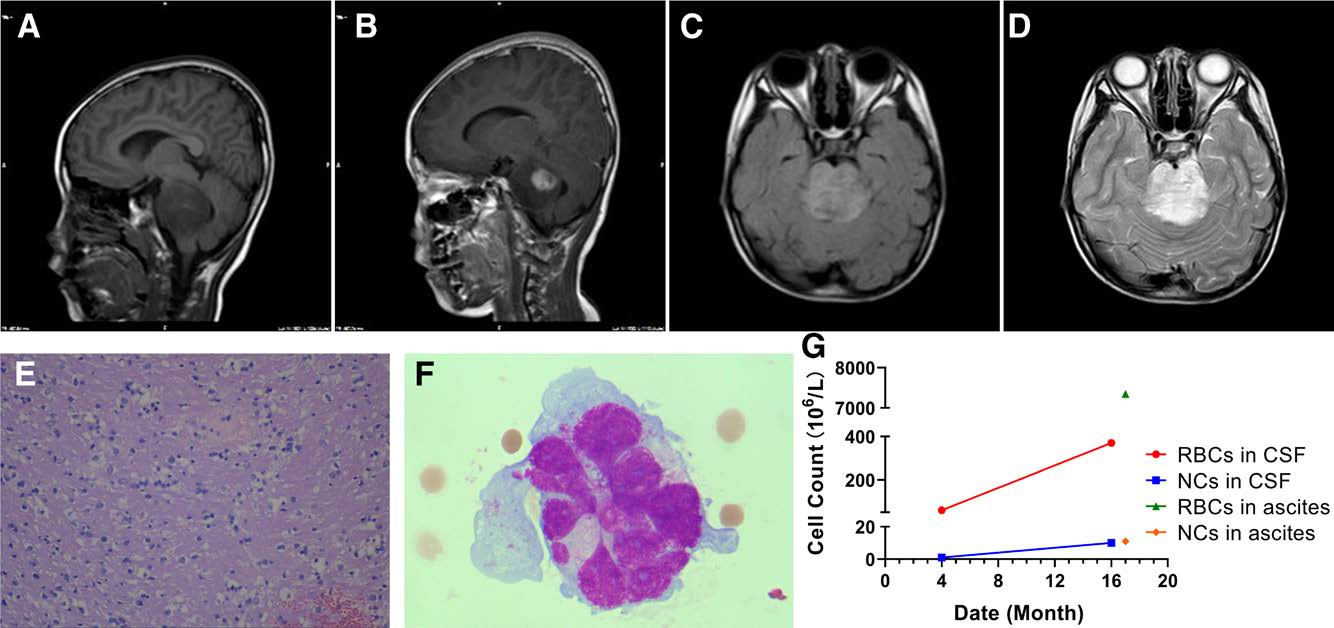
Case 2 (A) Sagittal T1 weighted image. (B) Sagittal T1 contrast-enhanced image. (C) Axial T1 weighted image. (D) Axial T2 weighted image. The lesion semi-circumscribed the adjacent basilar artery and mild postcontrast enhancing. (E) H&E of the cranial stereotactic biopsy. (F) Wright–Giemsa staining of the cells in CSF. (G) Trends of RBCs and NCs in CSF and ascites. CSF = cerebrospinal fluid, NCs = nucleated cells, RBCs = red blood cells.

A 5-year-old female presented with right lower limb weakness and blurred vision. Cranial MRI revealed significant swelling of the brainstem, with the lesion measuring approximately 49 × 35 × 41 mm, showing T1 hypointensity and T2 hyperintensity with heterogeneous enhancement (Fig. 2A–D). Histological examination confirmed a diagnosis of DMG with the H3 K27M mutation (Fig. 2E). The patient underwent multimodal treatment, including chemotherapy, local radiotherapy, targeted therapy (cabozantinib and piperacillin), and immune therapy (Ginsenoside Rg3 and oxytetracycline), which initially resulted in remission. However, 9 months post-biopsy, she developed symptoms of hydrocephalus, leading to the placement of a VPS and subsequent palliative radiotherapy at Zhujiang Hospital. Despite these interventions, complications arose 7 months later, including ascites and pneumonia. CSF analysis showed a significant increase in RBCs and NCs compared to baseline (Fig. 2G), with tumor cells detected in both CSF and ascitic fluid (Fig. 2F). Although chemotherapy was administered, the patient succumbed to central respiratory failure within 1 month of developing malignant ascites.

### 2.3. Case 3

**Figure 3.**
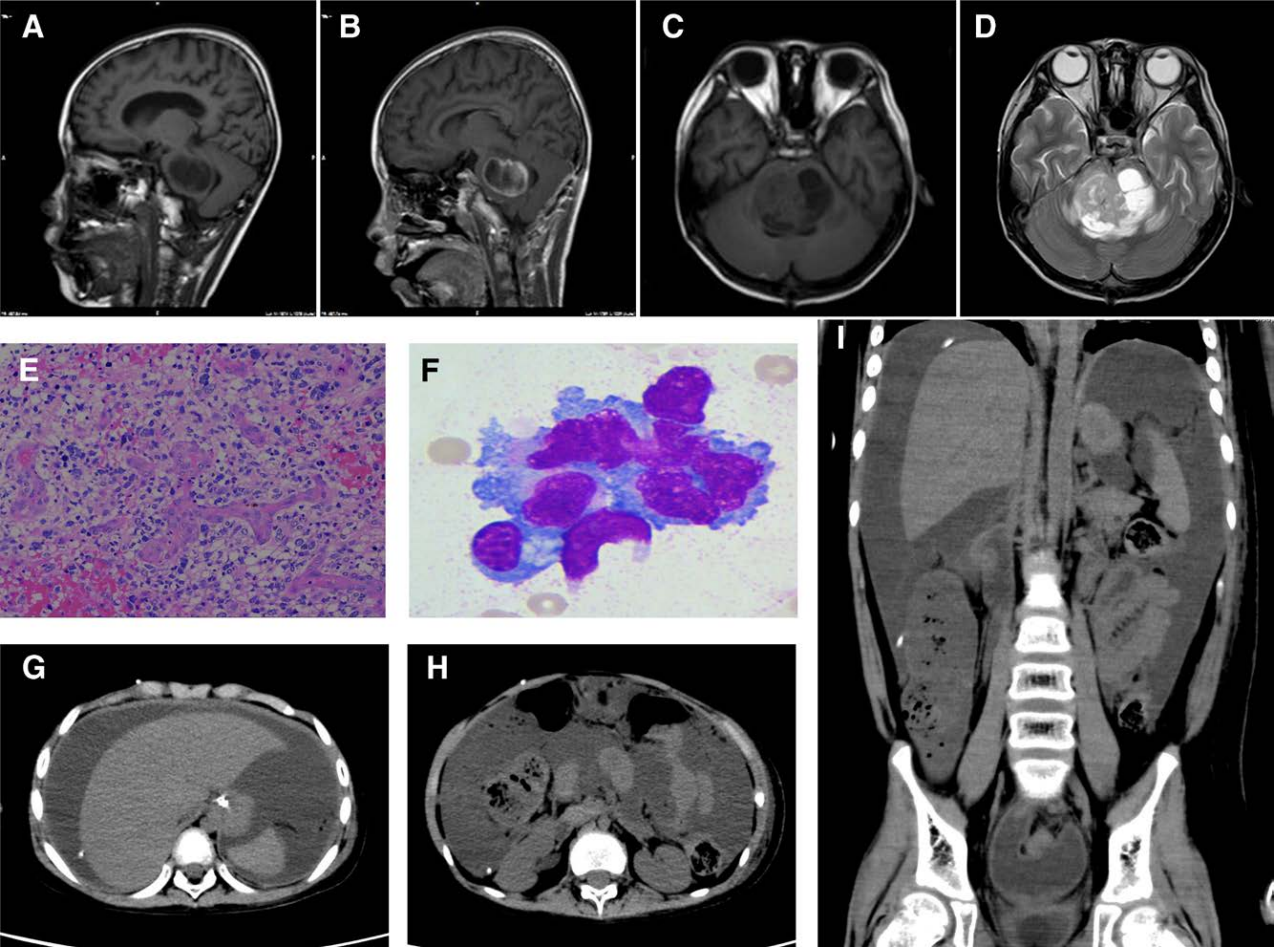
Case 3 (A) Sagittal T1 weighted image. (B) Sagittal T1 contrast-enhanced image. (C) Axial T1 weighted image. (D) Axial T2 weighted image. The lesion semi-circumscribed the adjacent basilar artery and mild postcontrast enhancing. (E) H&E of the cranial stereotactic biopsy. (F) Wright–Giemsa staining of the cells in ascites. (G–I) Abdominal and thoracic CT after VPS. CT = computed tomography, VPS = ventriculoperitoneal shunt.

An 8-year-old male presented to another hospital with generalized weakness and double vision. Cranial MRI revealed brainstem thickening, with a lesion measuring approximately 29 × 41 × 25 mm. The tumor had well-defined margins, appearing hypointense on T1 and hyperintense on T2, with cystic necrosis evident on contrast enhancement (Fig. 3A–D). Craniotomy was performed on the sixth day after diagnosis for partial removal of the brainstem lesion. Histopathological analysis confirmed cellular heterogeneity and presence of the DMG H3 K27M mutation (Fig. 3E). Owing to the development of hydrocephalus, the patient underwent right VPS placement 18 days after surgery at Shenzhen Children’s Hospital. Palliative radiotherapy in combination with bevacizumab was administered following family discussions, resulting in significant clinical improvement. However, 5 months after post-diagnosis (4 months after VPS placement), the patient was readmitted with abdominal distension and pain. Computed tomography revealed large amounts of fluid in the thoracic and abdominal cavities (Fig. 3G–I), with tumor cells present in the fluid (Fig. 3F). Chemotherapy was initiated for intra-abdominal metastasis; however, the disease progressed rapidly. The patient died within ten days of developing malignant ascites, with central respiratory failure being the direct cause of death.

## 3. Discussion

DMG is a rare and lethal tumor with a significantly worse prognosis in the presence of the histone H3.3 lysine-27-to-methionine (H3K27M) mutation,^[9]^ resulting in a median overall survival of approximately 12 months.^[10]^

This article presents 3 cases of DMG with H3K27M mutations. Each patient underwent a different treatment regimen: chemotherapy combined with gamma knife therapy (Case 1), chemotherapy with palliative radiotherapy (Case 2), and chemotherapy followed by surgical resection (Case 3). Despite these varying approaches, initial positive outcomes were observed. Notably, in Case 3, necrosis was effectively managed with bevacizumab, demonstrating satisfactory results consistent with the findings in the literature.^[11]^ However, owing to limitations in China’s health insurance, which only covers bevacizumab for tumors of the respiratory, digestive, and reproductive systems, its use for head tumors is not reimbursed. Consequently, after discussions with the patients’ families, bevacizumab was either omitted or used sparingly because of its high out-of-pocket costs. Supported by the literature, physicians are more inclined to try new antitumor drugs that are covered under insurance for head and neck tumors, such as nimotuzumab^[12,13]^and anlotinib,^[14,15]^ even though these drugs are not indicated for DMG in China. As the disease progressed, all 3 patients were readmitted, and a small number of heterogeneous cells were detected in the CSF (Table 1). This occurred 47 days post-VPS insertion in Case 1 and 438 days post-VPS insertion in Case 2, with no relevant tests conducted in Case 3. These heterogeneous cells, which are characteristic of various intracranial tumors, display specific features, such as large cell bodies, abundant cytoplasm, warty peripheral membranes, and intensely basophilic nuclei. Their presence in CSF indicates meningeal metastasis, which is an indicator of high sensitivity.^[16]^ Therefore, regular cytological analysis of the CSF in patients with intracranial tumors is crucial for reducing the risk of metastasis. This approach can be further enhanced by detecting circulating tumor DNA^[17]^ and proteins^[18]^ in the CSF, which aids in tracking tumor dissemination and changes in tumor burden,^[19]^ thus facilitating timely adjustments to treatment plans.

**Table 1:** Laboratory test of CSF and ascites in 3 children before and after shunt surger.

	Case 1			Case 2			Case 3	
	CSF		Ascites	CSF		Ascites	Ascites	
Point of time	1 d before VPS	47 d after VPS	149 d after VPS	70 d before VPS	438 d after VPS	460 d after VPS	96 d after VPS	98 d after VPS
RBCs(10^6^/L)	31	0	832	60	370	7349	1375	708
NCs (10^6^/L)	1	1	296	1	10	11	105	112
Heterotypic cell	−	+	+	−	+	+	+	+

CSF = cerebrospinal fluid, NCs = nucleated cells, RBCs = red blood cells, VPS = ventriculoperitoneal shunt.

While VPS insertion is commonly used for the treatment of hydrocephalus owing to its safety and convenience, certain complications inevitably arise from its long-term placement. The most frequent complications include infection,^[20]^ shunt dysfunction,^[21]^ and blockage,^[22]^ while shunt-related cysts and ascites are rare. These issues can occur at various intervals, ranging from 1 day to over ten years post-operation.^[23]^ We conducted a review of cases of intraperitoneal glioma metastasis associated with VPS (Table 2). The mean patient age was 5.1 years, with 3 males and 4 females. The VPS was chosen to alleviate hydrocephalus in all cases. However, despite the initial success, all patients were readmitted due to abdominal distension, and intra-abdominal metastasis was subsequently confirmed. In most cases (42%), the diagnosis was made via biopsy, consistent with current clinical practices, while in 2 cases, the diagnosis was confirmed through ascitic fluid aspiration.

**Table 2:** Reported cases of intra-abdominal metastasis of gliomas via ventriculoperitoneal shunts.

Age/sex	Tumour type	Time from VPS to IAM (mouth)	Symptoms of IAM	Confirmation of IAM	Treatment of IAM	Survival from IAM (mouth)	Reference
4/F	Pilocytic astrocytoma	5	Distension	Autopsy	Palliation	0.5	^[24]^
3/M	Anaplastic ependymoma	33	Distension	NS	CX	Alive	^[25]^
0.5/M	Pilocytic astrocytoma	2	Distension	Peritoneal aspirate	CX and XRT	Alive	^[26]^
7/M	Glioblastoma	7	Distension, emesis	Peritoneal aspirate	Palliation	1	^[24]^
15/F	Glioblastoma	25	Abdominal pain, distension	Biopsy	Palliation	6	^[27]^
4/F	DMG H3 K27M-mutant	12	Distension	Biopsy	XRT	1	^[28]^
2/M	Anaplastic gangliogliomas	1	Distension	Biopsy	XRT	1	^[8]^

CX = chemotherapy, DMG = diffuse midline glioma, IAM = intra-abdominal metastasis, NS = not stated, VPS = ventriculoperitoneal shunt, XRT = radiotherapy.

Endoscopic third ventriculostomy (ETV) is an emerging and effective technique for treating obstructive and selected cases of communicating hydrocephalus.^[29,30]^ Owing to permission constraints, we were unable to display the original preoperative MRI images in the main text. However, we contacted the 3 surgeons who performed the procedures, and they confirmed that all 3 patients exhibited varying degrees of dilation in the third and fourth ventricles along with aqueductal stenosis, consistent with a diagnosis of obstructive hydrocephalus. Despite this, in all 3 cases reported here, the surgeons opted for VPS insertion to relieve the hydrocephalus. Surgeons explained that VPS is a more commonly practiced and straightforward procedure, particularly in regions where proficiency in ETV is limited.^[31,32]^ Moreover, ETV has a higher failure rate than VPS, often necessitating a second surgery for permanent device placement following ETV failure.^[33]^ The choice of surgical method is influenced by regional practices, surgeon’s skill level, and patient’s clinical presentation. Both physicians and patients must be prepared for the outcomes of the selected surgical approach and potential sequelae that may follow.

Chemotherapy has shown promise in the literature, with 2 patients surviving post-treatment. However, in our reported cases, despite varying treatment approaches, all patients succumbed within a month of developing carcinomatous ascites, highlighting the need for further refinement of treatment. In 3 cases, ascites with glioma cells appeared 11, 7, and 5 months after VPS implantation, with causes extending beyond VPS complications. One possibility is tumor cell dissemination to the greater omentum, increasing its permeability and leading to malignant peritoneal effusion, which is commonly seen in cancers such as lymphoma^[34]^ and ovarian cancer.^[35]^ Although no space-occupying lesions were found, the presence of proliferating tumor cells in the peritoneal cavity supports intraperitoneal metastasis.^[36]^ Another possibility is CSF retention, where fluid pools in the peritoneal cavity through the shunt form the CSF ascites.^[37]^ Tumor cells that spread to CSF reabsorption sites, such as the leptomeninges and arachnoid membranes, could impair reabsorption and contribute to ascites. In summary, the causes of ascites post-VPS are multifactorial and require a thorough evaluation of patient symptoms to guide appropriate treatment.^[38]^

Few cases of extraneural dissemination of primary intracranial tumors have been reported, with most involving germ cell tumors and medulloblastomas.^[39,40]^ In contrast, extraneural dissemination of glioblastoma is rare, largely due to protective mechanisms, such as the blood-brain barrier, tumor-induced compression of veins, and the absence of cerebral lymphatic drainage at the dissemination site. However, surgical intervention can disrupt the blood-brain barrier and provide access to the lymphatic system. As a result, tumor cells may enter the cerebrospinal fluid and spread via extraneural pathways such as shunts, facilitating glioblastoma metastasis beyond the central nervous system.

**Figure 4.**
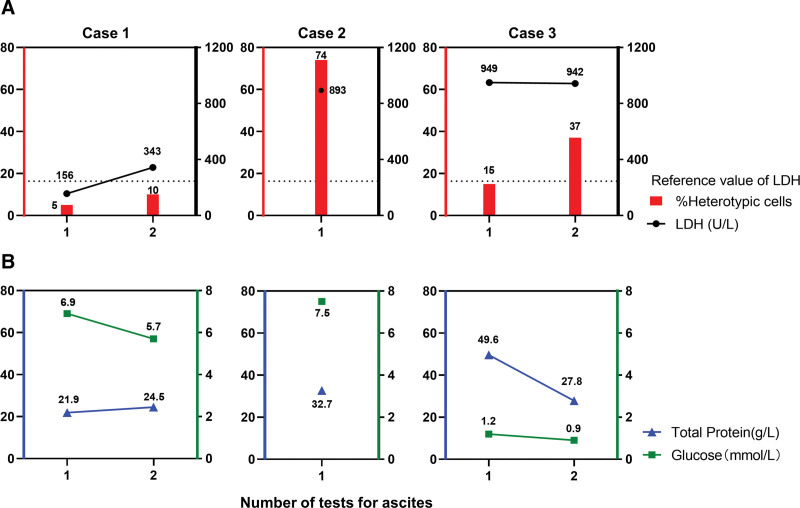
Trends of the proportion of isoform cells in ascites and biochemical indices of ascites in 3 children. (A) Trends in the proportion of isoforms in ascites and lactate dehydrogenase (LDH), the normal value of serum LDH should not be higher than 250 U/L. (B) Trends in the proportion of total protein (TP) and glucose (Glu) in ascites. Glu = glucose, LDH = lactate dehydrogenase, TP = total protein.

Biochemical analysis and cytological examination of the ascitic fluid revealed significant abnormalities, the elevated erythrocyte count in the ascites may have resulted from tumor cell invasion of the peritoneum. However, because of the children’s short survival period, no detectable abdominal metastases were observed. Lactate dehydrogenase (Fig. 4A) and total protein levels in the ascites were markedly elevated, while glucose levels in the ascites were lower than those in the blood (Fig. 4B), consistent with the exudative fluid characteristics. These findings suggest extensive tumor cell dissemination, with a high risk of infiltration. The conditions of all 3 patients deteriorated rapidly, and they succumbed within 1 month of developing carcinomatous ascites, consistent with biochemical indicators. Analyzing ascitic fluid components helps to elucidate the causes of ascites and the progression of the disease, providing valuable guidance for individualized treatment aimed at alleviating patient suffering.

This report presents 3 cases of DMG patients who developed ascites with tumor cells spreading to the peritoneal cavity following VPS implantation. Our findings underscore the critical role of CSF cytology in the early detection of glioma metastasis, and recommend regular monitoring of ascites to predict abdominal metastasis in patients with abdominal distension. However, this study was limited by the absence of preoperative MRI images of the VPS, which restricts our ability to further explore the potential of ETV as an alternative surgical approach. Additionally, due to the retrospective nature of this study, the patients were not routinely followed up at the hospital. Future research should include additional patient data to compare the prognostic outcomes of VPS versus ETV and implement regular follow-up visits to better assess the long-term outcomes.

## Author contributions

**Conceptualization:** Chunxia Huang, Shaoqiang Xu.

**Data curation:** Yuanyang Ye.

**Investigation:** Yunpeng Han, Keyuan Lai, Yuanyang Ye.

**Methodology:** Xubin Chai.

**Software:** Keyuan Lai.

**Supervision:** Yuanyang Ye.

**Visualization:** Chunxia Huang, Xubin Chai.

**Writing – original draft:** Chunxia Huang, Keyuan Lai, Shaoqiang Xu.

**Writing – review & editing:** Chunxia Huang, Xubin Chai, Yunpeng Han, Shaoqiang Xu.
